# Evidence for a synergistic effect of post‐translational modifications and genomic composition of eEF‐1α on the adaptation of *Phytophthora infestans*

**DOI:** 10.1002/ece3.7442

**Published:** 2021-03-18

**Authors:** Yan‐Ping Wang, E‐Jiao Wu, Yahuza Lurwanu, Ji‐Peng Ding, Dun‐Chun He, Abdul Waheed, Oswald Nkurikiyimfura, Shi‐Ting Liu, Wen‐Yang Li, Zong‐Hua Wang, Lina Yang, Jiasui Zhan

**Affiliations:** ^1^ Key lab for Bio pesticide and Chemical Biology Ministry of Education Fujian Agriculture and Forestry University Fuzhou China; ^2^ Department of Crop Protection Bayero University Kano Kano Nigeria; ^3^ School of Economics and Trade Fujian Jiangxia University Fuzhou China; ^4^ Fujian University Key Laboratory for Plant‐Microbe Interaction College of Life Sciences Fujian Agriculture and Forestry University Fuzhou China; ^5^ Institute of Oceanography Minjiang University Fuzhou China; ^6^ Department of Forest Mycology and Plant Pathology Swedish University of Agricultural Sciences Uppsala Sweden

**Keywords:** adaptation, compensatory evolution, housekeeping genes, methylation, plant pathogens, post‐translational modification, protein disordering, sustainable disease management

## Abstract

Genetic variation plays a fundamental role in pathogen's adaptation to environmental stresses. Pathogens with low genetic variation tend to survive and proliferate more poorly due to their lack of genotypic/phenotypic polymorphisms in responding to fluctuating environments. Evolutionary theory hypothesizes that the adaptive disadvantage of genes with low genomic variation can be compensated for structural diversity of proteins through post‐translation modification (PTM) but this theory is rarely tested experimentally and its implication to sustainable disease management is hardly discussed. In this study, we analyzed nucleotide characteristics of eukaryotic translation elongation factor‐1α (eEF‐lα) gene from 165 *Phytophthora infestans* isolates and the physical and chemical properties of its derived proteins. We found a low sequence variation of eEF‐lα protein, possibly attributable to purifying selection and a lack of intra‐genic recombination rather than reduced mutation. In the only two isoforms detected by the study, the major one accounted for >95% of the pathogen collection and displayed a significantly higher fitness than the minor one. High lysine representation enhances the opportunity of the eEF‐1α protein to be methylated and the absence of disulfide bonds is consistent with the structural prediction showing that many disordered regions are existed in the protein. Methylation, structural disordering, and possibly other PTMs ensure the ability of the protein to modify its functions during biological, cellular and biochemical processes, and compensate for its adaptive disadvantage caused by sequence conservation. Our results indicate that PTMs may function synergistically with nucleotide codes to regulate the adaptive landscape of eEF‐1α, possibly as well as other housekeeping genes, in *P. infestans*. Compensatory evolution between pre‐ and post‐translational phase in eEF‐1α could enable pathogens quickly adapting to disease management strategies while efficiently maintaining critical roles of the protein playing in biological, cellular, and biochemical activities. Implications of these results to sustainable plant disease management are discussed.

## Graphical abstract

We analyzed nucleotide characteristics of eukaryotic translation elongation factor‐1α (eEF‐lα) gene from 165 *Phytophthora infestans* isolates and the physical and chemical properties of its derived proteins. Our results indicate that post‐translation modifications may function synergistically with nucleotide codes to regulate the adaptive landscape of eEF‐1α, possibly as well as other housekeeping genes, in *P. infestans*. Compensatory evolution between pre‐ and post‐translational phase in eEF‐1α could enable pathogens quickly adapting to disease management strategies, while efficiently maintaining critical roles of the protein playing in biological, cellular, and biochemical activities.

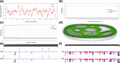

## 1 INTRODUCTION

Genetic variation, generated and maintained by interaction among evolutionary forces, plays a central role in pathogen adaptation to disease management schemes. High genetic variation increases the survival and reproductive potential of pathogen species because it enables the co‐existence of genotypes/phenotypes that can adapt to different environmental stresses induced naturally through climate changes or artificially through the deployment of disease management approaches. In nature, pathogen species differ markedly in the level of genetic variation generated by their respective evolutionary histories. Even genes coding for different biological and ecological functions within the same species may show significant difference in genetic/phenotypic variation. For example, mutations in genes critical to biological and/or ecological functions of species such as housekeeping genes greatly threaten survival and proliferation (Guibinga et al., 2010) and intend to be purged immediately by natural selection, leading to low genetic variation and a reduced ability to adapt to constant fluctuations in environmental stresses. Therefore, it is important to know what mechanisms the conserved genes in pathogen genomes have developed to counter this evolutionary disadvantage.

The complexity of genotype‐adaptation relationships arises from the fact that proteins have multiple structures. The primary structure of proteins encoded by gene sequences can be modified post‐transnationally, leading to different functional properties according to biological and ecological needs of a species. Post‐translational modification (PTM) can alter numerous properties of proteins including their catalytic activity, three‐dimensional structure as well as their interactions with other molecules, stability, and subcellular location. This modification capability is one of the important features of proteins in regulating cellular processes with many biological and ecological advantages (Mann & Jensen, 2003). For example, PTMs of proteins provide species an extended genetic variation that potentially empowers a rapid and precise response to environmental stresses without the fitness penalty associated with sequence changes at the pre‐translation stage. In cells, PTMs can be achieved in several ways, including methylation, formation of disulfide bridge, and alteration of ordering (Csizmok et al., 2016; Feige & Hendershot, 2011; Wu et al., 2017).

Protein methylation is created by adding one or several methyl groups to targeted proteins. Lysine, together with arginine, is one of the most methylated amino acids through which functional polymorphisms of proteins are generated (Lanouette et al., 2014). It is a common PTM event balanced by lysine methyl transferases and demethylases, and is involved in many cellular and biochemical processes critical to proliferation and adaptation of species. For example, methylation of lysine residues in eEF‐lα proteins is required for normal translational function and the extent of lysine methylation is positively correlated with the rate of protein synthesis in fungi (White et al., 2019). Therefore, we hypothesize that an over‐representation of lysine may exist in the proteins that required frequent methylation to promote functional diversity for natural adaptation.

A disulfide bridge is the covalent bond formed by linking sulfur atoms of two spatially close cysteine residues. This type of PTM can serve either as a structural or a biochemical role by affecting protein folding, stability, and function (Creighton et al., 1995; Xiao Liu et al., 2019; Zhang et al., 2018). There are three types of disulfide bonds in cells (Cook & Hogg, 2013). Catalytic disulfide bonds regulate thiol‐disulfide interchange reactions in substrate proteins, while structural disulfide bonds stabilize three‐dimensional arrangements of amino acids in proteins. On the other hand, allosteric disulfide bonds mediate functions of mature proteins by triggering conformational changes when they are cleaved or generated. These conformational changes can be related to ligand binding, substrate hydrolysis, proteolysis, and/or oligomer formation (Schmidt et al., 2006). Protein disulfide can occur in many cellular compartments such as endoplasmic reticulum, Golgi complex, and mitochondrial inter‐membrane space in eukaryotic species as well as the periplasmic space in bacteria (Braakman & Bulleid, 2011; Nakamoto & Bardwell, 2004) and faster accrual of disulfide bonds usually leads to greater functional diversity of proteins in species (Wong et al., 2011).

It has long been thought that a proper protein function relies on an ordered and stable structure. However, increasing number of studies has revealed that many biological and ecological important proteins are disordered and instable as they do not have a well‐defined three‐dimensional arrangement (Uversky, 2015). Disordered protein or disordered protein regions usually contain a large proportion of hydrophilic amino acids (Williamson & Potts, 2012) and a lack of disulfide bonds (Ghag et al., 2017). The physical characteristics of disordered proteins ensure their readiness for further PTM and a capacity for conformational changes, interaction surface expansion, and the generation of molecular recognition elements (i.e. short linear interaction motifs‐SLiMs) to promote interactions with other proteins and molecular compounds (Moritsugu et al., 2012). Disordered proteins are more prevalent in higher species and are particularly enriched in the biological and biochemical processes associated with cell‐signaling transduction, DNA transcription, protein translation, and chromatin remodeling events (Romero et al., 2004). It has been found that disordered proteins can facilitate the adaptation of species to new environments and species among ecological niches differed in the extent of protein disordering (Schlessinger et al., 2011), suggesting an evolutionary advantage to this type of PTM.

Evolutionary theory proposes that PTMs work synergistically with the genomic composition of genes to shape the genetic adaptation of species, but empirical data to support this hypothesis are lacking. In this study, we tested this theory by a jointing analysis of sequence characteristics of the eukaryotic translation elongation factor‐1α (eEF‐1a) gene in *Phytophthora infestans* and the physical, chemical, cellular, and signal transduction properties of its derived protein. *Phytophthora infestans* is one of the most notorious plant pathogens worldwide. It has caused significant economic and sociological impacts including the Great Irish famine (Yoshida et al., 2013) and remains the main constraining factor of potato and tomato industries (Kröner et al., 2017). EEF‐1α is responsible for delivering aminoacyl‐tRNAs to ribosome in a GTP‐mediated reaction during translation and the second most abundant protein after actin. It accounts for 1%–2% of the total protein in normal growing cells and the abundance of eEF‐1α in cells is correlated with levels of protein synthesis and proliferation (Condeelis, 1995; Mateyak & Kinzy, 2010). EEF‐1α is highly methylated and stably expressed during biotic and abiotic stress in plants (Hiatt et al., 1982; Nicot et al., 2005). In addition, the eEF‐1α protein is believed to have other functions such as regulating the actin cytoskeleton (Munshi et al., 2001), promoting viral replication (Li et al., 2014), and is involved in signal transduction, transformation, and immunoreactivity (Piedra‐Quintero et al., 2015; Skarina et al., 2011). For example, removal of the eEF‐1α protein predisposed cells to be more susceptible to malignant transformation in soybean (Tatsuka et al., 1992).

Therefore, the specific objectives of the study are to: (a) investigate sequence variation of the eEF‐1α gene in *P. infestans*; (b) characterize the primary, secondary, and tertiary structure of the eEF‐1α protein; (c) predict the chemical property, subcellular localization, and interacting networks of the eEF‐1α protein; and (d) infer the contribution of post‐translation modifications to the evolutionary adaptation of conserved genes such as eEF‐1α in *P. infestans*. Understanding these questions would provide us fresh insights into the evolutionary mechanisms of housekeeping genes as well as ways of formulating effective and sustainable strategies of ameliorating disease impacts to crops and human society under future climate condition.

## 2 MATERIALS AND METHODS

### 2.1 Pathogen collection and genotyping

*Phytophthora infestans* isolates were sampled from 11 potato fields located in Heilongjiang, Inner Mongolia, Ningxia, Shanxi, Gansu, Henan, Hubei, Chongqing, Guizhou, Guangdong, and Guangxi provinces and one tomato field located in Fujian province, China. These 11 regions represent the most intensive potato production areas in the country (Jansky et al., 2009), while tomato cultivation in Fujian has expanded steadily in the past decades due to increasing demand and improved cultivation techniques. During the epidemic seasons, potato and tomato leaves with late blight symptoms were collected at 1–2 m intervals from infected plants and transported to the laboratory within 24 hr for *P. infestans* isolation. After cleaning with running tap water and sterilized distilled water, infected leaves were placed abaxial side up on 2.0% water agar medium in the dark for ~24 hr at 18°C. *Phytophthora infestans* strains were isolated by inoculating a fraction of mycelia tipped from a sporulating lesion onto a rye B Petri dish using an inoculating needle. Strains were purified by three serial transfers of mycelia to fresh rye B media. Details of the pathogen collection and isolation are described in previous publications (Qin et al., 2016; Wang et al., 2020). Only one isolate was secured from each infected leaf and a total of 1,473 isolates were secured from the samples.

To produce mycelium mass for DNA extraction, the purified *P. infestans* isolates were cultured in the dark on rye B agar at 18°C for 15 days. Harvested mycelia were transferred into 2‐ml sterile centrifuge tubes and lyophilized with a vacuum freeze‐dryer (Alpha1‐2, Christ, Germany) for 6 hr. The lyophilized mycelia were ground to powder with a mixer mill (MM400, Retsch, Germany). Total DNA of *P. infestans* was extracted using a plant genomic DNA kit (Promega Biotech. Co. TRANSGEN. China) according to the manufacturer's instructions. The genomic DNA was suspended in 50 μl deionized water and stored at −40°C until used. Genotypes of the isolates were determined by the software GENCLONE 2.0 (Arnaud‐Haond & Belkhir, 2007) using the data generated from molecular amplification of SSR markers (Knapova & Gisi, 2002; Lees et al., 2006), restriction enzyme‐PCR amplification of mitochondrial haplotypes (Flier et al., 2003), mating type (Zhu, Vossen, et al., 2015; Zhu, Yang, et al., 2015), and partial sequence analysis of b‐tubulin, Cox1, and Avr3a (Martha et al., 2011). For isolates with the identical genotype, only one of them was selected for sequence analysis of the eEF‐1α gene. As a result, 165 *P. infestans* isolates each with a distinct genotype were included in the current study. Among them, 156 genotypes were from potato and nine isolates from tomato. These genotypes differed in two to six markers from each other.

### 2.2 *Phytophthora infestans* eEF‐1α Sequencing

DNA extracted from the 165 genetically distinct *P. infestans* isolates was amplified using a pair of eEF‐1α‐specific primers (F: 5′‐ GCCATATACAGCTGAGAAATCTCA‐3′ and R: 5′‐ CTGTACAGTAGATGAGAATCAGATG ‐3′). PCR amplifications of the eEF‐1α gene were performed in a total reaction volume of 50 μl composed of 1.0 μl HifiTaq DNA polymerase, 5.0 μl 10 × HiFi Buffer II, 4.0 μl of dNTPs (10 μmol/L), 2.0 μl of forward primer (10 μmol/L), 2.0 μl of reverse primer (10 μmol/L), 34 μl of ddH2O, and 2.0 μl of template DNA using a Gene CyclerTM (Bio‐Rad). Amplifications started with a DNA denaturation step at 95°C for 5 min; followed by 35 cycles of amplification at 94°C for 30 s, annealing at 61°C for 30 s, and extension at 72°C for 1.5 min; and ended with a further extension step at 72°C for 5 min. PCR products were separated on 1% agarose gels by electrophoresis and purified for single direction sequencing according to manufacturer's instructions (QIAquick^®^ Gel Extraction Kit). The purified products were ligated into a T1 zero cloning vector and transformed into Trans1‐T1 competent cells by heat‐shock at 42°C for 30 s (pEASY^®^‐T1 Zero Cloning Kit). Colonies with single and expected amplicon size were sequenced by GenScript Biological Technology Co., Ltd. (GenScript, Nanjing, China) using an ABI3730 automated DNA sequencer (Applied Biosystems, USA). Details of purification and sequencing are described in previous publications (Wang et al., 2020; Yang et al., 2020).

### 2.3 Fitness tests of *Phytophthora infestans*

Fitness of the 165 genetically distinct genotypes was estimated by measuring their in vitro growth rate, tolerances to thermal stress, fungicide and UV irradiation and aggressiveness. Unless specifically defined, these experiments were conducted at 19°C, the optimum temperature for the pathogen, with three replicates and parameters were measured at the sixth day after inoculation using the image analysis software Assess (Lamari, 2002). In vitro growth rate was estimated by colony size formed on rye B agar. Thermal, fungicide, and UV tolerances were measured by the colony size of the isolates formed under 25°C, in the presence of azoxystrobin (0.15 μg/ml) or exposed to UV radiation (300 s, wavelength = 254 nm) relative to that formed under 19°C, in the absence of the fungicide or without UV irradiation, respectively. For these measurements, colonies were initiated by taking a mycelia plugs (*ϕ* = 3 mm) from revived isolates and inoculated on rye B media supplemented with Ampicillin (100 mg L^−1^) and Rifampin (10 mg L^−1^) in 9‐cm Petri dishes. Aggressiveness of the isolates was tested on the susceptible potato cultivar Bintje by the detached leaflet assay (Zhu, Vossen, et al., 2015; Zhu, Yang, et al., 2015). In this assay, fully expanded potato leaflets were drop‐inoculated with sporangial suspensions and kept on 2% water‐agar plates in an incubator supplemented with 16h light daily and diseased areas of the leaflets were measured at seven days after inoculation. The detailed protocols for the measurements of in‐vitro growth rate, tolerance to thermal stress, fungicide and UV irradiation and aggressiveness can be found in previous publications (Lurwanu et al., 2021; Wu et al., 2019; Yang et al., 2016, 2019).

### 2.4 Data analysis

Nucleotide sequences were visually assessed to remove fake “mutations” caused by PCR artifacts (Yang et al., 2013). Amino acid isoforms were deduced from the nucleotide sequences by MEGA 7.0.21 (Sudhir et al., 2016). Nucleotide and amino acid compositions in the eEF‐1α were analyzed using BioEdit Sequence Alignment Editor (Hall, 1999), and homogeneity of nucleotide proportions in the gene was evaluated by chi‐square test (Kathleen et al., 2017). Nucleotide haplotype network was constructed by a maximum parsimony approach embedded in DnaSP 6 (Rozas et al., 2017) and displayed by PopArt version 1.7 (Leigh & Bryant, 2015). Recombination events were evaluated by the algorithms implemented in the RDP4 suite (Martin et al., 2015). The PROFsec algorithm embedded in an online tool Predicted Protein (http://www.predictprotein.org) was used to construct and annotate the secondary structure of eEF‐1α proteins. ESPript 3.0 (http://espript.ibcp.fr/ESPript/ESPript/) was used to compute secondary structure information of eEF‐1α proteins and the structural similarities between the proteins. The 3D structure of the eEF‐1α protein was modeled according to the crystal structure of the yeast elongation factor complex eEF1A:eEF1BA (Jank et al., 2017) using the SWISS‐MODEL via the ExPASy web server (https://www.expasy.org/). Model accuracy was evaluated by Global Model Quality Estimation (GMQE). Hydrophobicity of eEF‐1α proteins was evaluated by a Kyte & Doolittle (K‐D) approach (Kyte & Doolittle, 1982) embedded in the BioEdit version 7.1.3.0 program (Hall, 1999) and transmembrane domains of the eEF‐1α proteins were predicted using the online program TMHMM 2.0 (http://www.cbs.dtu.dk/services/TMHMM/). The online program SignalP version 5.0 (http://www.cbs.dtu.dk/services/SignalP/) was employed to forecast the signal peptides of the eEF‐1α proteins based on combined deep convolutional and recurrent neural network architecture and conditional random field approaches, TargetP version 1.1 (http://www.cbs.dtu.dk/services/TargetP/). LocTree3 approach was used to predict the subcellular location of the eEF‐1α protein and produce a cell schematic diagram (Goldberg et al., 2012). DISULFIND (Ceroni et al., 2006) embedded in Predicted Protein was used to predict disulfide bridges in the eEF‐1α protein. Predicted Protein was also applied to predict protein‐protein and protein‐polynucleotide binding sites using the ProNA2020 function (Qiu et al., 2020) and to evaluate the protein ordering based on PROFbval, Ucon and Meta‐Disorder (MD) functions (Schlessinger et al., 2006, 2007, 2009). Potential short linear motifs (SLiMs) were predicted by ANCHOR (http://anchor.elte.hu/). STRING version 11.0 (https://string‐db.org/), an online tool searching for retrieval of interacting genes, was used to assess and integrate the interaction networks of the eEF‐1α with other proteins. The protein‐protein interaction (PPI) network was visualized by Cytoscape software version 3.7.2 (Shannon et al., 2003). SMART (http://smart.embl.de/) was used to predict the functional domains within the eEF‐1α protein. Analysis of variance for in vitro growth rate, tolerances to thermal stress, fungicide and UV irradiation and aggressiveness was performed by the general linear model procedure (GLM) and difference between eEF‐1α isoforms in these parameters was evaluated by least significant difference (LSD) using SAS version 9.1.3.

## 3 RESULTS

### 3.1 Sequence characteristics of *Phytophthora infestans* eEF‐1α gene and protein

**FIGURE 1:**
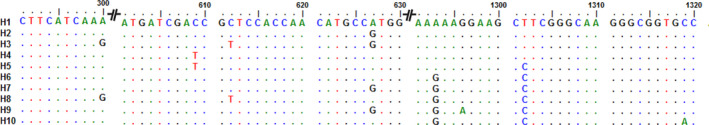
Multiple sequence alignment of 10 *Phytophthora infestans* eEF‐1α nucleotide haplotypes sampled from tomato and potato. Dots indicate identical nucleotides with H1 sequence in the top

**FIGURE 2:**
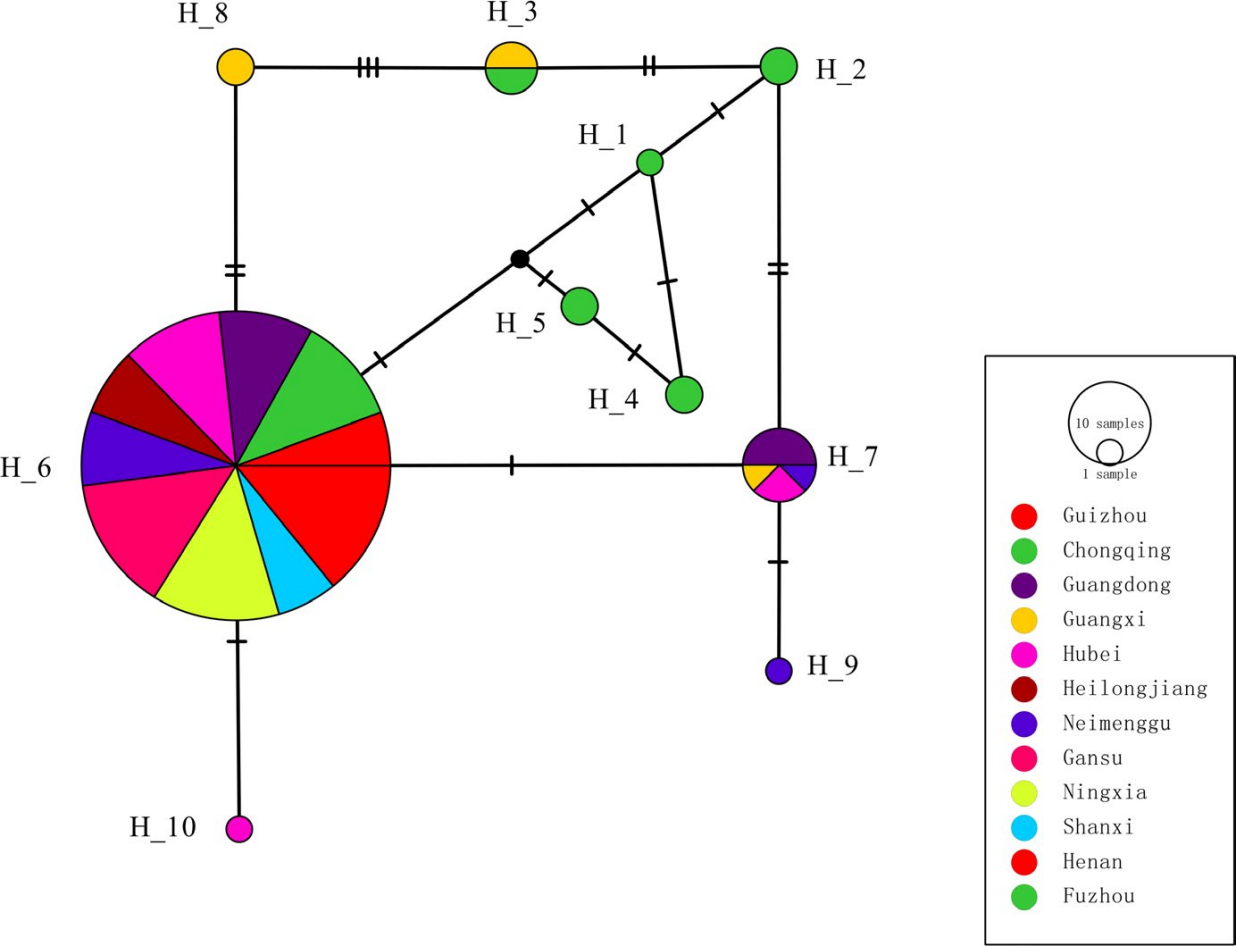
Nucleotide haplotype network of eEF‐1α gene of *Phytophthora infestans* populations sampled from 11 potato and 1 tomato planting area in China. The network was constructed by a maximum parsimony approach. Nucleotide haplotypes are named by the letter H followed by a corresponding number. Each circle represents a unique haplotype and size of circles indicates the frequency of isolates with that particular haplotype. Each tick mark represents a step of nucleotide substitution. Black circles represent missing haplotypes

Full eEF‐1α sequences were generated from all 165 genetically distinct *P. infestans* isolates included in the study and deposited in GenBank with accession numbers of MN422761‐MN422925. The full eEF‐1α gene had 1,332 nucleotides, translated into a protein with 443 amino acids in length. A total of 10 nucleotide haplotypes (Figure 1) were identified from the 165 sequences, with four privates to tomato (H1, H2, H4, H5) and five privates to potato (H6‐H10). Only one haplotype (H3) was shared among the two hosts. The 10 haplotypes were connected to H6, the most common haplotype by 1–5 mutation steps (Figure 2). Although three reticulation structures existed in the haplotype network, no recombination events were detected (data not shown). Haplotype and nucleotide diversity of the eEF‐1α gene were 0.89 and 0.0017 from tomato, which are higher than 0.17 and 0.0002 from potato, respectively. However, only two amino acid isoforms (I‐1 and I‐2) were deduced from the 165 nucleotide sequences. All except two nucleotide sequences (H9, H10) were translated to the dominant isoform I‐1. These two sequences were detected from potato in two different locations and had a mutation from alanine to aspartic acid at the 440th amino acid, generating the minor isoform I‐2. Isoform I‐1 had a significantly higher in vitro growth rate, aggressiveness and tolerance to thermal stress, fungicide and UV irradiation than isoform I‐2 (Table 1).

**TABLE 1:** Least significant difference in fitness between *Phytophthora infestans* isolates with eEF‐lα isoform I‐1 and I‐2

Isolates	In vitro growth rate (cm^2^)	Tolerance			Aggressiveness (cm^2^)
		Temperature (25°C)	Azoxystrobin (0.15μg/mL)	UV (300s, 254 nm)	
Iso‐1	2.77 ± 0.04A^a^	0.692 ± 0.0010A	0.335 ± 0.007A	0.893 ± 0.015A	6.17 ± 0.08A
Iso‐2	2.41 ± 0.02B	0.574 ± 0.006B	0.255 ± 0.003B	0.752 ± 0.011B	5.52 ± 0.05B

^a^ Values followed by different letters in within a column differ significantly at *p* = .01.

**FIGURE 3:**
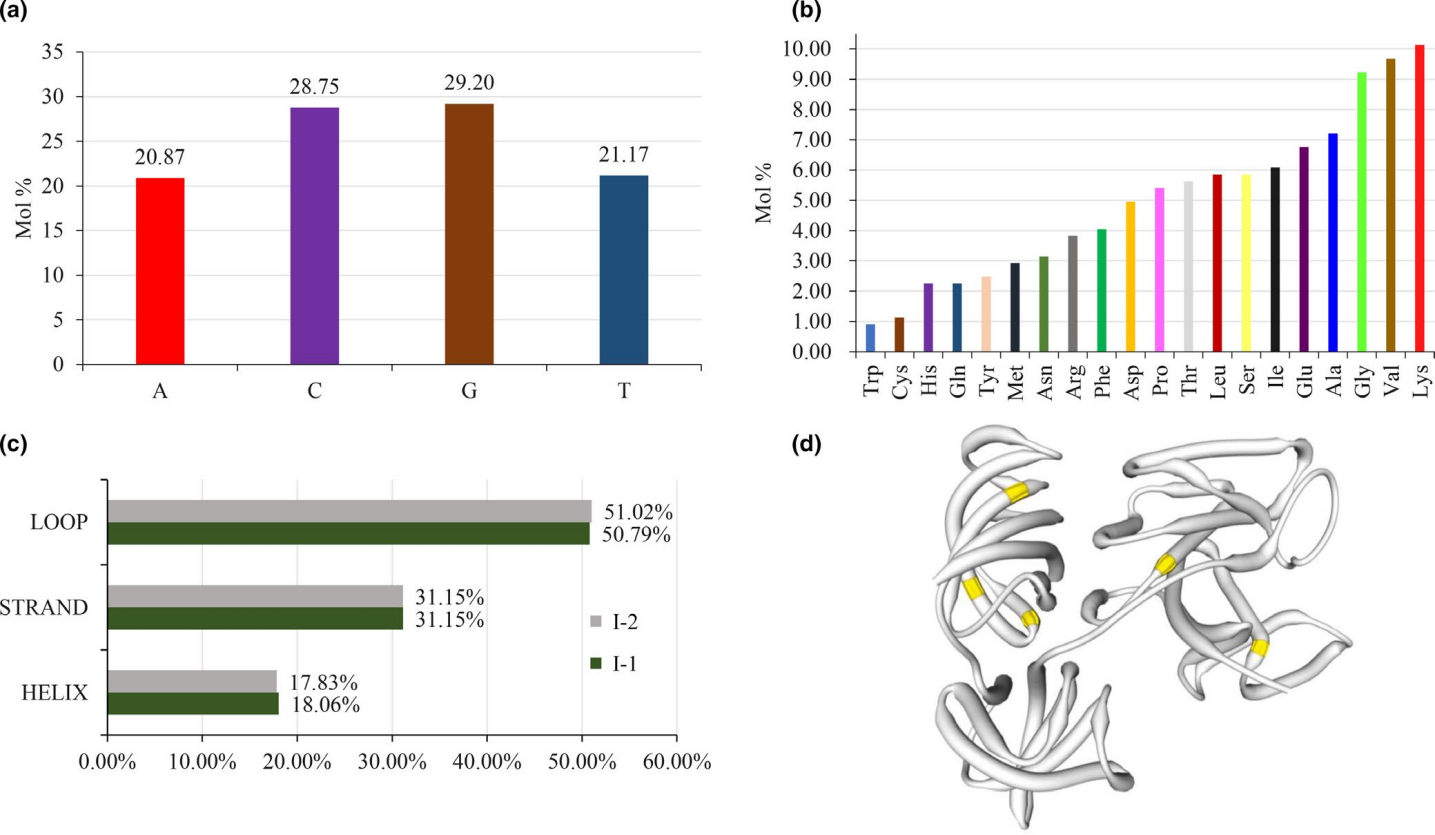
Molecular and structural characteristics of eEF‐1α in the *Phytophthora infestans* population from China. (A) nucleotide composition; (B) amino acid composition; (C) secondary structure of the eEF‐1α proteins; and (D) 3D model computed by SWISS‐MODEL based on the crystal structure of the yeast elongation factor complex eEF1A:eEF1BA. Yellow represents the positions of cysteine

The average percentage of A, C, G, and T in the eEF‐1α nucleotide haplotypes translated to I‐1 was 20.87%, 28.75%, 29.20%, and 21.17% (20.95%, 28.68%, 29.20%, and 21.17% for I‐2), respectively (Figure 3a). Further analyses showed that base composition in the gene significantly deviated from the theoretical expectation of equal proportion (*p* < .0001) and GC content was significantly higher than AT content (*p* < .0001). The two isoforms contained all 20 basic amino acids ranging from 0.90% for tryptophan to 10.14% for lysine (Figure 3b). I‐2 had one less alanine but one more aspartic acid than I‐1 had. The five cysteine residues in the two isoforms were located at amino acid positions 31, 111, 351, 358, and 388, respectively. There were no disulfide bonds in the tertiary structure of the protein.

**FIGURE 4:**
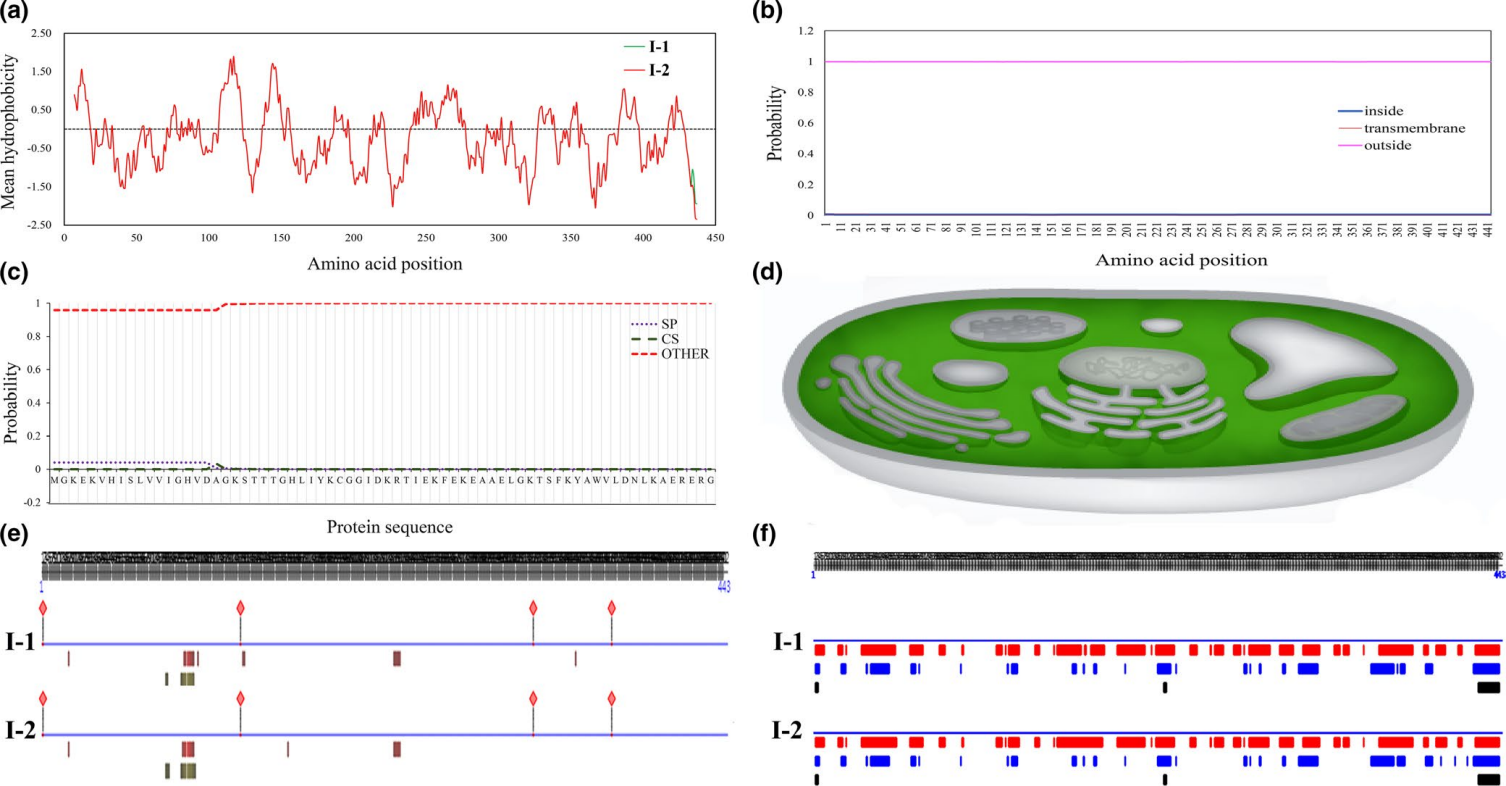
The basic properties of eEF‐1α proteins in *Phytophthora infestans* sampled from potato and tomato: (A) hydrophobicity estimated by a Kyte & Doolittle (K‐D) approach embedded in the BioEdit version 7.1.3.0 program; (B) prediction of transmembrane domains using TMHMM 2.0; (C) inference of signal peptides by SignalP version 5.0 program based on a combination of deep convolutional and recurrent neural network architecture and conditional random field; (D) subcellular localization (green) of the proteins; (E) protein‐protein (pink diamonds), protein‐macromolecule (red rectangles) and RNA‐macromolecule (green rectangles) binding sites predicted by ISIS or ProNA2019 method; and (F) protein disorder predicted by PROFbval (red), Ucon (blue) and Meta‐Disorder (black)

Secondary structure analysis showed that both I‐1 and I‐2 contained three motifs: a Helix (18.06% and 17.83%), a β‐strand (31.15% and 31.15%), and a ω‐loop (50.79% and 51.02%), respectively (Figure 3c). The 3D structure of the *P. infestans* eEF‐1α protein was identical to the yeast elongation factor in the 5o8w.1.A complex with >95% confidence (Figure 3d). The eEF‐1α protein contained more hydrophilic regions than hydrophobic regions (Figure 4a) and was a cytoplasmic protein (Figure 4d) due to a lack of trans‐membrane helices and signal peptides (Figure 4b,c). I‐1 and I‐2 shared protein‐protein and RNA‐macromolecule binding sites but differed in protein‐macromolecule binding sites. Four protein‐macromolecule binding sites (102nd, 131st, 132nd, and 347th amino acids) were predicted in I‐1, while only one unique protein‐macromolecule binding site (amino acid 160) was detected in I‐2 (Figure 4e). Many short linear interaction motifs (SLiMs) were detected in the eEF‐1α proteins. PROFbval and Meta‐Disorder (MD) methods predicted that I‐1 and I‐2 shared all disordered motifs but the Ucon method showed that mutation in the 440th amino acid of I‐1 generated several disordered regions, which formed a new SLiM between amino acid 421 and 427 in I‐2, predicted by ANCHOR software (Figure 4f).

### 3.2 Protein‐protein interaction (PPI) and domain analysis

**FIGURE 5:**
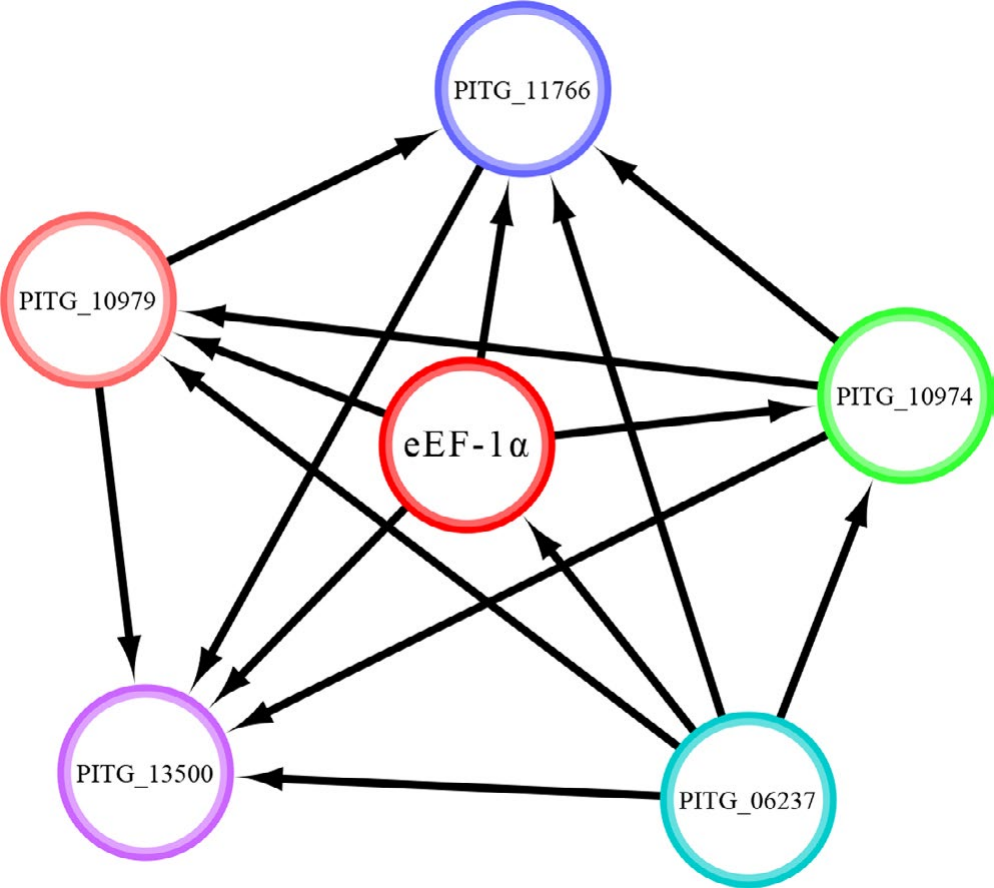
The protein–protein interaction (PPI) network of *Phytophthora infestans* eEF‐1α constructed by STRING using the information extracted from public databases. *P. infestans* eEF‐1α protein (Gene symbol: PITG_06722) is a highly identical to I‐1 and I‐2. PITG_10979, PITG_10974, PITG_06237, PITG_11766, and PITG_13500 are candidate elongation factor 1γ and ribosomal protein of *P. infestans* (Table 1)

**FIGURE 6:**
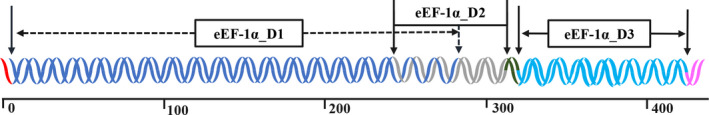
SMART predicted three Pfam domains (eEF‐1α_D1, eEF‐1α_D2, and eEF‐1α_D3) in the eEF‐1α protein of *Phytophthora infestans*. Among them, eEF‐1α_D1 and_D2 share a 36 amino acid overlapping region

Results from STRING analysis showed that I‐1 and I‐2 sequences were highly similar (>99%) to PITG_06722, a *P. infestans* elongation factor 1‐alpha protein that promotes the GTP‐dependent binding of aminoacyl‐tRNA to the A‐site of ribosome during protein biosynthesis. A 6‐node network varying in strength of connectivity was built (Figure 5) based on neighborhood, fusion, co‐occurrence, co‐expression, texting, and homology of proteins built in STRING library. The top five hub proteins including two elongation factor 1 gamma (putative) proteins (PITG_10979 and PITG_10974) and three ribosomal proteins (PITG_06237, PITG_11766, and PITG_13500, Table 2) had a higher degree of connectivity with eEF‐1α of the current study. SMART analysis revealed three Pfam domains located between the 5th and 284th, 248th and 315th, and 321st and 430th amino acid in the eEF‐1α proteins with high levels of confidence (Figure 6, Table 3). The eEF‐1α_D1 domain, a typical structure of GTP‐dependent proteins that can bind non‐initiator tRNAs and ribosomes together, was highly similar to both EF‐1α/EF‐Tu of prokaryotes and EF‐2/EF‐G of eukaryotes. The eEF‐1α_D2 domain had 36 amino acids overlying the eEF‐1α_D1 domain in the N‐terminal and adopted a beta‐barrel structure to bind with charged tRNA (PUBMED: 7491491). The domain was structurally related to the C‐terminal domain of EF2 in eukaryotes and archaea (IPR004160) and other proteins such as translation initiation factor IF‐2 and tetracycline‐resistance proteins of bacteria. The eEF‐1α_D3 domain is located at the C‐terminal of the eEF‐1α proteins and has adopted a beta‐barrel structure to bind charged tRNA with EF1B ([(PUBMED: 9253415) or EF‐Ts, IPR001816).

**TABLE 2:** Descriptions and scores of the 10 candidate *Phytophthora infestans* proteins interacting with eEF‐1α

Accession no.	Protein description	Score
PITG_10979	Elongation factor 1‐gamma, putative (367 aa)	0.989
PITG_10974	Elongation factor 1‐gamma, putative (406 aa)	0.989
PITG_06237	60S ribosomal protein L8, putative (256 aa)	0.962
PITG_11766	40S ribosomal protein S3a; Belongs to the eukaryotic ribosomal protein eS1 family (261 aa)	0.952
PITG_13500	40S ribosomal protein S4; Belongs to the eukaryotic ribosomal protein eS4 family (261 aa)	0.949
PITG_09345	40S ribosomal protein S5–2; Belongs to the universal ribosomal protein uS7 family (196 aa)	0.941
PITG_20264	40S ribosomal protein S13; Belongs to the universal ribosomal protein uS15 family (151 aa)	0.938
PITG_02578	Ribosomal protein L15; Belongs to the eukaryotic ribosomal protein eL15 family (206 aa)	0.936
PITG_15697	60S ribosomal protein L11, putative; Belongs to the universal ribosomal protein uL5 family (133 aa)	0.935
PITG_03221	40S ribosomal protein S18; Belongs to the universal ribosomal protein uS13 family (152 aa)	0.931

Note

The information was retrieved from public databases by STRING.

**TABLE 3:** SMART prediction of eEF‐1α domains and their amino acid locations in *Phytophthora infestans*

Name	Start	End	*E*‐value
Pfam: eEF‐1α_D1	5	284	6.00E−54
Pfam: eEF‐1α_D2	248	315	1.40E−14
Pfam: eEF‐1α_D3	321	430	7.10E−44
low complexity	431	443	

## 4 DISCUSSION

Low genetic variation was found in the eEF‐1α gene of *P. infestans*. Only two amino acid isoforms were identified in the 10 nucleotide haplotypes generated from 165 isolates. The observed low genetic variation is consistent with the theoretical expectation for housekeeping genes (Zhang & Li, 2004) where reduced evolution can ensure functional conservation critical to the survival, proliferation and therefore fitness of species (Scaggiante & Bosutti, 2015) including pathogens. We hypothesize that the lower genetic variation is caused by purifying selection (Viscidi & Demma, 2003) coupled with a lack of recombination rather than a reduced mutation rate in the gene and we have two lines of evidence to support this argument. First, haplotype network analysis indicates the nucleotide haplotypes (H9 and H10) translated to minor isoform I‐2 are in the tips of tree and are likely descended from the nucleotide haplotypes translated to the major isoform I‐1 (Figure 2). Biological tests show that isolates with mutant isoform I‐2 were mal‐adapted to temperature, UV and fungicide stresses and caused less late blight disease on potato compared to the dominant, parental isoform (Table 1), suggesting that the non‐synonymous mutation in the eEF‐1α gene may reduce the fitness of *P. infestans* and be selected against. SMART analysis revealed a 36 amino acid overlap between the eEF‐1α D1 and D2 domains (Figure 6). This structure further reduces the tolerance of the eEF‐1α gene to non‐synonymous mutations (Gussow et al., 2016). Although the number of I‐2 isolates involving in fitness tests is not optimum and future studies should strike to include more such isolates if possible, we believe that the observed fitness difference between the two isoforms unlikely caused by rest of genome. In one hand, the two I‐2 isolates differ from each other in mating type and several SSR loci and originate from different locations, therefore, are unlikely to be the same clonal lineage. On the other hand, they are genetically more closed to dominant Chinese isolates than some of the isolates with isoform I‐2. Furthermore, the four measurements of fitness in the study are quantitative traits likely controlled by genes randomly distributing across the genome.

Second, although there are a low number of amino acid isoforms, as many as 10 nucleotide haplotypes and some reticulation structures were detected in the haplotype network (Figure 2). Intra‐gene recombination was not detected in both the current or previous sequences (Wang et al., 2020) in the eEF‐1α gene and in the eEF‐1α gene the reticulation structures are likely caused by convergent mutations. Intra‐gene recombination events have been detected in effector and fungicide target genes of the same pathogen collection (Chen et al., 2018; Yang et al., 2020; Yang et al., 2018). Third, In particular, five nucleotide haplotypes were detected in the nine isolates from tomato. These results suggest that mutations have occurred frequently in the eEF‐1α gene. It has been documented that genome context can have an impact on the mutation rate of a species and mutation rates in genes with high GC contents tend to be elevated (Kiktev et al., 2018). Indeed, significantly higher GC than AT content was found in the eEF‐1α gene (Figure 3a).

Lysine accounts for 10.14% of the amino acids in the eEF‐1α protein of *P. infestans* (Figure 3b). This is ~50% higher than the theoretical expectation estimated from codon frequency (http://www.tiem.utk.edu/~gross/bioed/webmodules/aminoacid.htm), and a similar trend was also found in the eEF‐1α proteins of other species (Figure S1). The lysine content in eEF‐1α proteins of *P. infestans* was also higher than other genes such as RXLR and CRN effectors in the species (Figure S2). Lysine is a particular target for PTM events including acetylation, methylation, phosphorylation, ubiquitination, and sumoylation (Azevedo & Saiardi, 2016). Among these PTMs, methylation represents the most complex and common event occurred in lysine and up to three methyl groups can be added to the ε‐amine of lysine residues for the methylation (Lanouette et al., 2014). Meanwhile, the extent of lysine N‐methylation of eEF‐lα is correlated with the rate of protein synthesis in eukaryotes such as fungus, rabbit, and human (Jakobsson et al., 2017; Sherman & Sypherd, 1989). Higher than expected lysine content in the eEF‐lα sequences of *P. infestans* and other species provides the protein a greater opportunity to modify its functions during bio‐cellular and biochemical processes either through methylation or other PTM events. This enhances the adaptation of species to environmental and ecological stresses such as the adaptation of pathogens to changing climatic conditions and deployment of disease management strategies in agricultural systems. Indeed, it has been documented that eEF‐lα is among a few non‐histone proteins with lysine methylation and ~20% of lysine residues in the protein are methylated during translation in some species (Sherman & Sypherd, 1990). This result suggests that the evolutionary disadvantage of the eEF‐lα protein associated with low genetic variation at the pre‐translation level may be compensated for by its structural and functional plasticity at the post‐translation level and is consistent with evolutionary theory that postulates that protein compositions can work synergistically with the genetic codes to determine the adaptive landscape of species (Beltrao et al., 2013) including pathogens.

Disulfide bond metabolism is governed by the frequency and distribution of cysteine in the relevant proteins (Rietsch and Beckwith, 1998). Cysteine representation in the cells is correlated positively with the complexity of organisms, ranging from 0.5% in Archaebacteria to 2.26% in mammals. In our study, we found that cysteine accounted for 1.13% of the amino acid composition in the eEF‐1α protein, which is within the expected range for the species, but no disulfide bonds were detected. In most organisms, disulfide bonds are formed by jointing the thiol groups of cysteines located two amino acids apart (Pu et al., 2018). In the eEF‐1α protein of *P. infestans,* the five cysteines are at amino acid 31, 111, 251, 358, and 388. The long spatial distance between cysteine residues may contribute to the lack of disulfide bonds in the eEF‐1α protein. This lack of disulfide bonds reduces the stability of the eEF‐1α protein, which is consistent with the analysis that shows the elongation protein is highly disordered in *P. infestans* (Figure 4f) and some other species (Ramesh & Sattlegger, 2020; Soares et al., 2009; Yang et al., 2020).

In comparison with proteins with well‐defined structures, disordering proteins or protein regions possess several functional advantages including increases in the interaction surface and conformational flexibility to interact with other compounds (Babu et al., 2011) for cellular processes or pathogenicity of pathogens (Yang et al., 2020). During translation, eEF‐1α needs to bind with several other macromolecules such as ribosomal proteins, RNAs, and polymerases. Usually, the ability and efficiency of these interactions are positively associated with the loop length of proteins (Papaleo et al., 2016). In the eEF‐1α protein of *P. infestans*, the loop does not differ from the normal length (50%) of other proteins (Figure 3c). The disordered regions enhance the eEF‐1α protein's ability and efficiency to form translation complex with other essential macromolecules (Table 2, Figure 5) and to produce proteins and enzymes required for the survivals, reproduction and pathogenicity of pathogens. These results further indicate that protein compositions, through their impacts on potential of PTMs such as protein ordering, could work together with genome structure to compensate for the evolutionary disadvantage of conserved genes such as eEF‐lα and other housekeeping genes in *P. infestans* in species. Indeed, it has been documented that protein disordering and many other PTMs can escalate the adaptation of species including pathogens to new environments independent of nucleotide variation (Brown et al., 2011; Nilsson et al., 2011; Volkwein et al., 2019; Xu et al., 2020; Yang et al., 2020).

In addition to protein biosynthesis, it has been reported that eEF‐1α is also involved in a wide range of other biological and biochemical processes (Mateyak & Kinzy, 2010) as a consequence of its interact with SAM domain and HD domain‐containing protein 1 (SAMHD1), phospholipase C gamma 1 (PLCG1), actins, muscarinic and acetylcholine receptors, the poly(A)‐binding protein (PABP1) and many others in both cytoplasm and nucleus (Chang et al., 2002; Vera et al., 2014). Interestingly, cellular localization and co‐expression analyses of protein–protein interaction indicate that the eEF‐1α of *P. infestans* only existed in the cytoplasm and did not interact with other proteins except ribosomal proteins and elongation factors (Table 2, Figure 5). Biochemical analyses also found that the eEF‐1α is a hydrophilic protein without trans‐membrane helices (Figure 4a,b,c), preventing its involvement in mRNA transportation during protein synthesis as documented in mammals (Vera et al., 2014). These results suggest that the function of the eEF‐1α protein in *P. infestans* might be largely constrained to translation only but this hypothesis needs to be verified experimentally by functional analyses of the genes.

Functional conservation in housekeeping genes such as eEF‐1α is critical for survival, proliferation, and adaptation of species. Fast evolution of pathogens associated with shorter generation times relative to their hosts is a beneficial to genes involving in host‐pathogen arms race but may generate genetic loads to housekeeping genes. Compensatory evolution between pre‐ and post‐translational phase in eEF‐1α empowers pathogen's ability to quickly adapt to disease management strategies, while efficiently maintain the critical roles of the housekeeping genes playing in the biological, cellular, and biochemical activities of the pathogens. To achieve sustainable disease management strategies should be formulated using all available arsenals including quarantine, disease forecast, and primary inoculum eradiation and the conserved feature of eEF‐1α and possibly other housekeeping genes would be a valuable resource for accurate and fast pathogen detections, which are unexclusive components of the quarantine, disease forecast, and inoculum eradiation processes.

## CONFLICT OF INTEREST

None declared.

## AUTHOR CONTRIBUTIONS

**Yan‐Ping Wang:** Data curation (lead); formal analysis (lead); validation (lead); visualization (lead); writing–original draft (lead); writing–review and editing (lead). **E‐Jiao Wu:** Data curation (equal); formal analysis (equal); validation (supporting); visualization (equal); writing–review and editing (supporting). **Lurwanu Yahuza:** Data curation (equal); formal analysis (equal); writing–review and editing (supporting). **Ji‐Peng Ding:** Data curation (supporting); formal analysis (supporting); writing–review and editing (supporting). **Dun‐Chun He:** Data curation (supporting); formal analysis (supporting); writing–review and editing (supporting). **Abdul Waheed:** Data curation (supporting); formal analysis (supporting); writing–original draft (supporting); writing–review and editing (supporting). **Oswald Nkurikiyimfura:** Formal analysis (supporting); visualization (equal); writing–review and editing (supporting). **Shi‐Ting Liu:** Data curation (supporting); visualization (supporting); writing–original draft (supporting). **Wen‐Yang Li:** Data curation (supporting); visualization (supporting); writing–review and editing (supporting). **Zong‐Hua Wang:** Supervision (equal); validation (supporting); writing–review and editing (supporting). **Li‐Na Yang:** Conceptualization (equal); formal analysis (supporting); funding acquisition (equal); project administration (equal); supervision (equal); visualization (supporting); writing–original draft (equal); writing–review and editing (equal). **Jiasui Zhan:** Conceptualization (lead); funding acquisition (lead); project administration (equal); supervision (lead); validation (supporting); writing–original draft (equal); writing–review and editing (equal).

## Supporting information

Fig S1

Fig S2

## Data Availability

Associated gene sequences data were deposited in Genbank with accession numbers of MN422761–MN422925.
