# Supplementary figures and images for: Evidence for a synergistic effect of post‐translational modifications and genomic composition of eEF‐1α on the adaptation of Phytophthora infestans
Source: Ecol Evol. 2021 Mar 18;11(10):5484–96. doi: 10.1002/ece3.7442 (PMC8131795; doi:10.1002/ece3.7442)

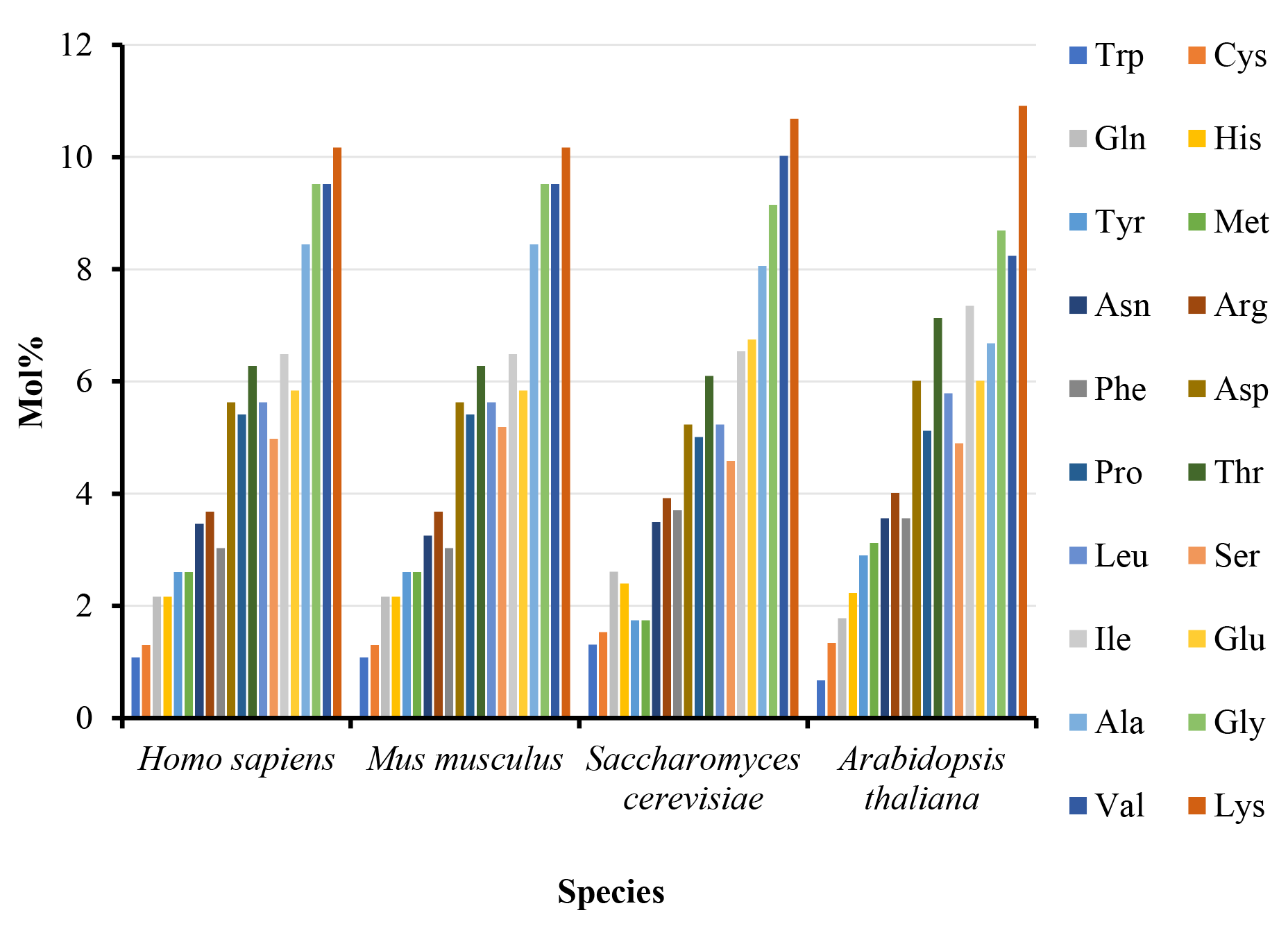

Supplement: Supplementary file 1 — Fig S1 [file ECE3-11-5484-s001.tif]

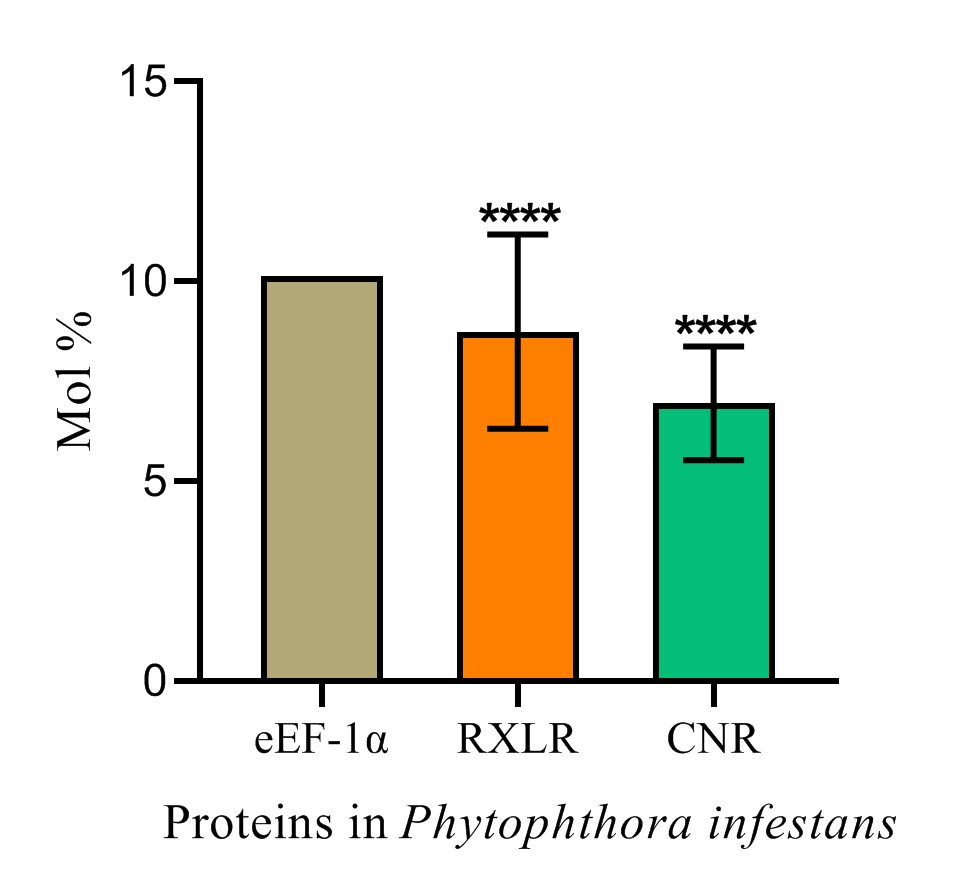

Supplement: Supplementary file 2 — Fig S2 [file ECE3-11-5484-s002.tif]
